# Effects of the depletion of neural progenitors by focal X-ray irradiation on song production and perception in canaries

**DOI:** 10.1038/s41598-023-36089-1

**Published:** 2023-06-02

**Authors:** Ioana Chiver, Ednei B. dos Santos, Shelley Valle, François Lallemand, Charlotte A. Cornil, Gregory F. Ball, Jacques Balthazart

**Affiliations:** 1 GIGA Neurosciences, University of Liege, 15 Avenue Hippocrate, 4000 Liège, Belgium; 2 Cyclotron Research Center, University of Liege, 4000 Liège, Belgium; 3 Department of Psychology, University of Maryland, College Park, MD 20742 USA

**Keywords:** Physiology, Systems biology, Neuroscience, Neural circuits, Neurogenesis, Zoology, Animal behaviour

## Abstract

The song control nucleus HVC of songbirds has emerged as a widespread model system to study adult neurogenesis and the factors that modulate the incorporation of new neurons, including seasonal state, sex differences or sex steroid hormone concentrations. However, the specific function of these new neurons born in adulthood remains poorly understood. We implemented a new procedure based on focal X-ray irradiation to deplete neural progenitors in the ventricular zone adjacent to HVC and study the functional consequences. A 23 Gy dose depleted by more than 50 percent the incorporation of BrdU in neural progenitors, a depletion that was confirmed by a significant decrease in doublecortin positive neurons. This depletion of neurogenesis significantly increased the variability of testosterone-induced songs in females and decreased their bandwidth. Expression of the immediate early gene ZENK in secondary auditory areas of the telencephalon that respond to song was also inhibited. These data provide evidence that new neurons in HVC play a role in both song production and perception and that X-ray focal irradiation represents an excellent tool to advance our understanding of adult neurogenesis.

## Introduction

Adult brain plasticity takes multiple forms^1,2^. In the song control system of songbirds, morphological plasticity can be observed based on measures such as the overall volume of the individual song nuclei, cell size and spacing of neurons, complexity of the dendritic branching, and adult neurogenesis in HVC (used as a proper name). In mammals, new neurons are also incorporated in the adult brain primarily in the hippocampus and olfactory bulbs^3,4^. But the rate of this neurogenesis observed in mammals is less active by orders of magnitude than in birds. It only concerns a few hundred new neurons per day^5^ whereas male canaries yearly replace about 50% of HVC neurons that project to RA, the robustus nucleus of the arcopallium (up to 50 to 100,000 new neurons from one season to the next)^6,7^. The replacement rate reaches at its peak of about 1.4% of HVC neurons every day^8^.

Studies have identified the site of production of new neurons and their fate from the division of neural progenitors to mature neurons incorporated into functional circuits (reviewed in^5,8–11^). The rate of adult neurogenesis varies across the annual cycle in temperate zone birds such as canaries. These changes correlate with the annual changes in singing behavior^7,12,13^ and are largely controlled by testosterone^14,15^.

The specific function of the new HVC neurons resulting in changes in HVC overall volume is poorly understood. Initial work suggested that larger HVC volumes mediate increased song repertoire^16^. This view was later challenged by negative results^17–19^ and it was proposed that HVC volume (and possibly rate of neurogenesis) rather relates to the amount of singing (reviewed in^20^). HVC neurogenesis is thus correlated to singing activity or song quality (e.g.^21^). Several studies (reviewed in^22^) have more specifically related neurogenesis to song stability but all this work is essentially correlational and work testing in a causal manner the function of new HVC neurons has been scarce. For example, targeted photolysis of HVC neurons that project to RA affected the rate of new neuron recruitment and song quality but effects on song quality were ambiguous: a detectable song deterioration was observed only in four of the nine treated birds^23^.

Analysis of neurogenesis function is difficult and relies on a limited number of tools. One of them involves injection of antimitotic drugs such as arabinofuranosyl cytidine (AraC) or methylazoxy-methanol acetate (MAM) that block cell divisions. However, these compounds have side effects, that have been characterized in mammals mostly, and have rarely been used in birds (see however^24,25^). Genetic methods are now being developed to induce apoptosis in specific cell types in mice via targeted injections of a virus expressing a toxin receptor or caspase-3 and a consensus element directing the action to a specific cell type (e.g.^26,27^). Some viruses are also able to specifically ablate neural progenitors and immature dentate granule cells in the mouse hippocampus (e.g.^28^). Implementation of these techniques in avian systems remains technically difficult and to our knowledge, they have never been used to study the role of adult neurogenesis in HVC of songbirds.

Focal irradiation (with gamma or X-rays) of neurogenic zones in the brain has also been used in mammals^29–33^. We are not aware of any study that has employed this approach to study HVC neurogenesis. We established here the feasibility of this irradiation approach and then investigated the functional consequences of the irradiation of HVC and the adjacent neurogenic zone on the development of testosterone-induced singing in females.

## Methods

### Subjects

Two experiments were performed with canaries (*Serinus* *canaria*) of the Fife fancy strain. The Fife fancy canary, a breed selected for posture, is a miniature version of the Border that has retained a clear sensitivity to changes in photoperiod. Males in this breed sing proficiently but their song, although quite diversified, is slightly less elaborate than in the German (Harz) roller or the Wasserslager that were selected for singing.

For experiment 1 that was essentially a proof of concept, eight males were obtained from a colony at University of Antwerp, Belgium, in March 2019. They were approximately 3 years of age at the beginning of the experiment in August. Birds were maintained in groups of 2–3 males per cage on a 10L:14D light regime. For experiment 2, 14 females were obtained from a local breeder at approximately 18 months of age in March 2019 and kept in a large (2.5 m × 2.5 m surface and 3 m tall) indoor aviary at University of Liege until the start of the experiment in April 2021 when they were transferred to individual sound-attenuated boxes. In the aviary, birds were in an all-female group of approximately 30 females and had access to visual and auditory stimuli from males (varying from 5 to 30 males) of the same strain kept in separate cages in the same room. These birds were maintained on an 8L:16D light regime at ambient temperatures between 19 and 24 °C both in the aviary and during the experiment.

Fresh seeds and water were provided ad libitum along with anise-scented shells and water bath to all birds under all conditions. All animal procedures complied with Belgian laws regarding the Protection and Welfare of Animals and the Protection of Experimental Animals and were approved by the University of Liege Animal Use and Care Committee (protocol number 2027). This study was carried out in compliance with the ARRIVE guidelines.

### General design

Experiment 1 used male subjects with large HVCs and an established full song to demonstrate the effectiveness of the X ray for depleting neural progenitors at the target. To this end, irradiation was only unilateral in order to provide an internal control. This approach indeed allowed testing in a more stringent manner the anatomical specificity of the irradiation and its effects by comparing the irradiated and non irradiated side. Since irradiation was only unilateral, little or no change in singing behavior was anticipated. We nevertheless recorded and analyzed songs before the irradiation and just before brain collection. This confirmed the absence of a qualitatively important deterioration of songs but since no control non-irradiated group was included, no firm conclusion could be obtained and these results will not be presented here. Brains were collected two weeks after the irradiation, which represents the peak of arrival of new neurons in HVC^34,35^.

In experiment 2, the main goal was to assess functional deficits following bilateral irradiation. Females were used because they rarely sing, but a high rate of singing behavior can be induced by exogenous testosterone^36–39^. It was thought that song induction would be more sensitive to the depletion of new neurons than an already established singing behavior. Since females do not sing much spontaneously and testosterone takes about 4 weeks to produce most of its activating effects on song^38,40^, brain were only collected after 4 weeks to optimize chances of observing some behavioral deficits. This ensured that control females had enough time to develop a full song and that the decreased neurogenesis in irradiated birds would result in a substantial cumulative depletion of new neurons.

**Figure 1:**
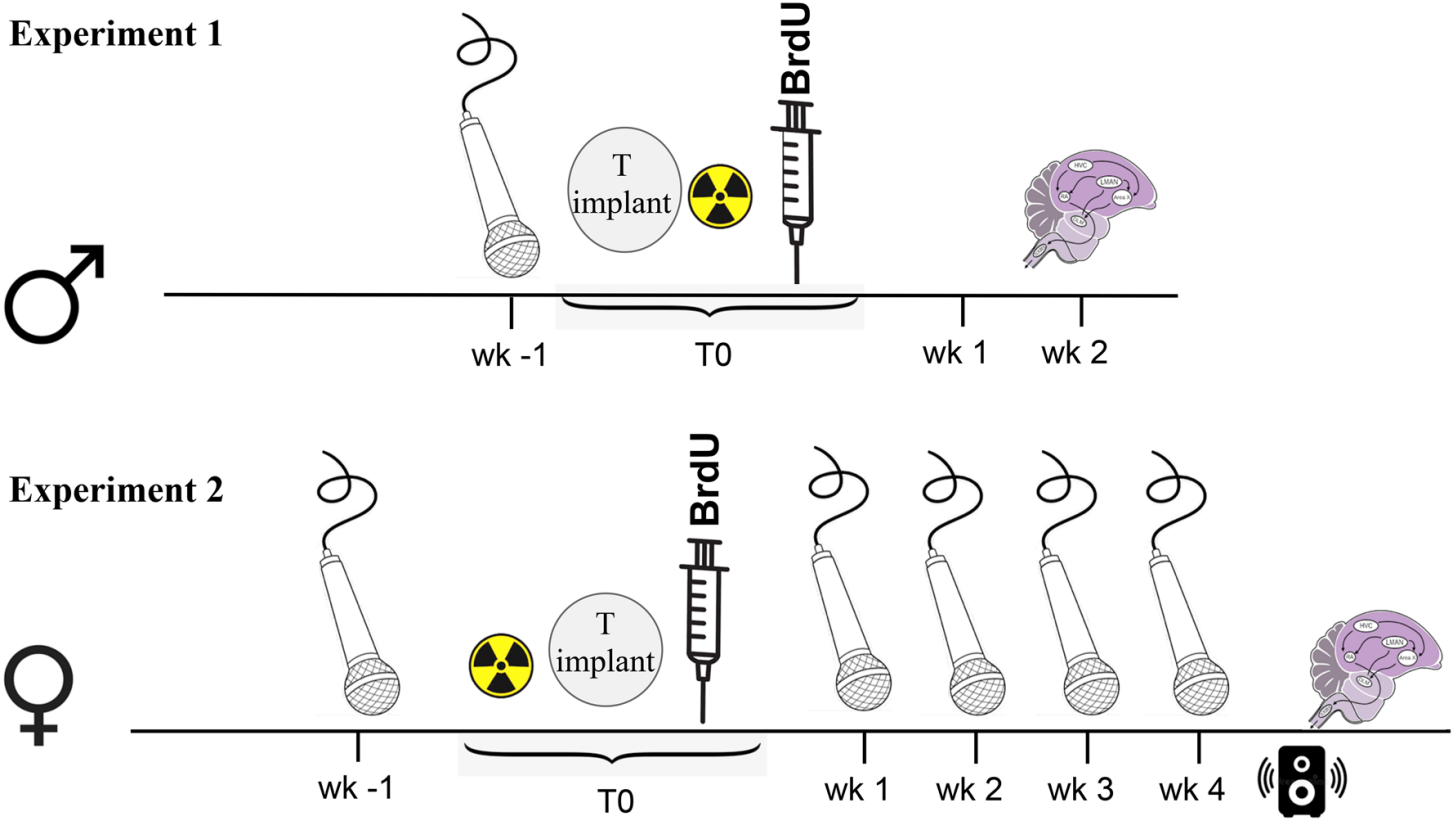
Schematic representation of the experimental timeline with the different manipulations performed during experiments 1 and 2, including song recordings (microphone), irradiation of the ventricular zone dorsal to HVC (radioactive sign, unilateral in experiment 1, bilateral in experiment 2), BrdU injection (syringe), song playback (loudspeaker) and brain collection (sagittal brain drawing). T0: time 0 i.e., beginning of treatments; wk1–4: weeks 1 to 4.

Prior to experimental manipulations, birds were moved to individual sound-attenuated boxes fitted with microphones that allowed individual recording of song. Treatment involved unilateral or bilateral irradiation for experiment 1 and 2 respectively, implantation with testosterone, and BrdU injections to monitor neurogenesis (Fig. 1). Song characteristics were determined from recordings in the week prior to treatment and assessed during the four weeks following treatment in experiment 2.

### Irradiation of progenitor cells in ventricular zone dorsal to HVC

It has become clear that neural progenitors are more sensitive than mature neurons to the effects of ionizing irradiation. These radiation treatments cause DNA breakup and, as a consequence, disruption of protein synthesis and of specific biochemical pathways. Immature rapidly dividing cells are therefore likely to be affected more profoundly. By exposing proliferating precursors to a limited dose of radiation (range 10 to 25 Gray), it is thus possible to selectively kill them without (extensive) collateral damage (see^29^ for a detailed discussion of this specificity question). In addition, this irradiation can easily be focused to the zone of interest, which further increases the specificity of effects. This approach was used here in two separate experiments.

At the onset of experiment 2 there was a delay due to COVID and to technical problems that made the irradiator non-functional. Females thus remained in the boxes for 8 weeks before the start of the experiment. Each subject was recorded for 2 h from 8–10 AM to confirm the low vocal output typical in females (experiment 2). Birds were then randomly divided in two groups matched as far as possible on the vocal features recorded during the pre-experimental recordings.

Irradiation of the ventricular zone dorsal to the HVC was performed either unilaterally (experiment 1) or bilaterally (experiment 2) using a small animal irradiator and scanner (Small Animal Radiation Therapy with advanced precision, SmART; Instrument from Precision X-Ray North Branford, CT, USA) designed to image, target and irradiate cells and small animals up to the size of rats (see https://precisionxray.com/small-animal-igrt/).

The bird was placed on a platform inside the irradiator with the beak inserted into a 10 mL syringe fixed to the platform and connected to the isoflurane vaporizer. Anesthesia was induced using 3% isoflurane for the first 30 s after which it was lowered to 1.5–2% for maintenance. First, a pre-scan of the head was obtained to image the brain and locate the target area of the ventricular zone dorsal to HVC. The scanner uses a cone-beam computed tomography that involves rotating 360° around the head of the bird to collect approximately 400 images that are then used to re-construct a 3D image of the target area. The target irradiation field was then delineated using the irradiator software and this target was irradiated with a dose of 23 Grays that was determined by the software based on the irradiation time, number of irradiating beams, distance from the source and volume of the target area.

**Figure 2:**
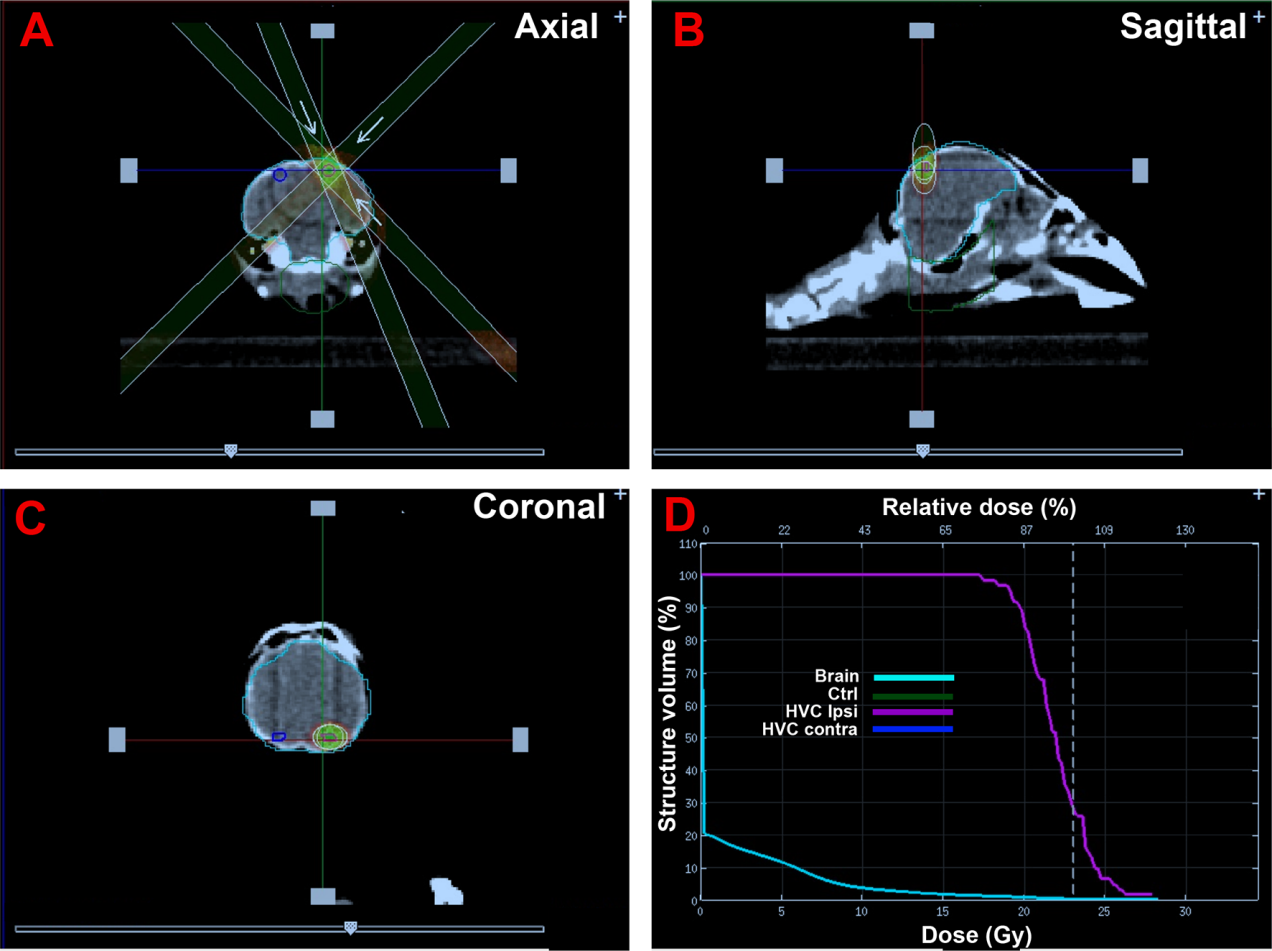
Selected copies of panels of the irradiator computer screen showing the localization of the three X-ray beams focused on the ventricle dorsal to the right HVC. (**A–C**) Illustrate the focalization of these beams in the axial, sagittal and coronal planes. (**D**) Illustrates the distribution of the energy in the target area (HVC ipsilateral to irradiation in pink), in the rest of the brain (light blue), in the non-irradiated contralateral HVC (dark blue) and in a control region placed on the cerebellum (dark green, visible in (**A**) only). The areas that are concerned are circled in panels (**A–C**) in the corresponding color. Note that the contra-lateral HVC and the control area (cerebellum) did not receive any detectable energy so that the corresponding curves do not appear in (**D**). About 90% of the X-ray dose was actually administered to the targeted HVC (**D**). The 3 beams approximately delivered the same dose of irradiation, 7.59, 7.81 and 7.71 Gy (total 23.0 Gy) over a period of 210, 361 or 290 s for beams 1 to 3 respectively.

In experiment 1, the dose of 23 Grays was distributed among 3 beams focused on the target in the ventricular zone dorsal to HVC (see Fig. 2). Males were assigned randomly to have either the left (n = 4) or right (n = 4) hemisphere irradiated. With this approach, most of the irradiating energy accumulated in the target area (pink line in panel D) and the limited amount of energy hitting the rest of the brain (light blue) was insufficient to cause significant damage.

**Figure 3:**
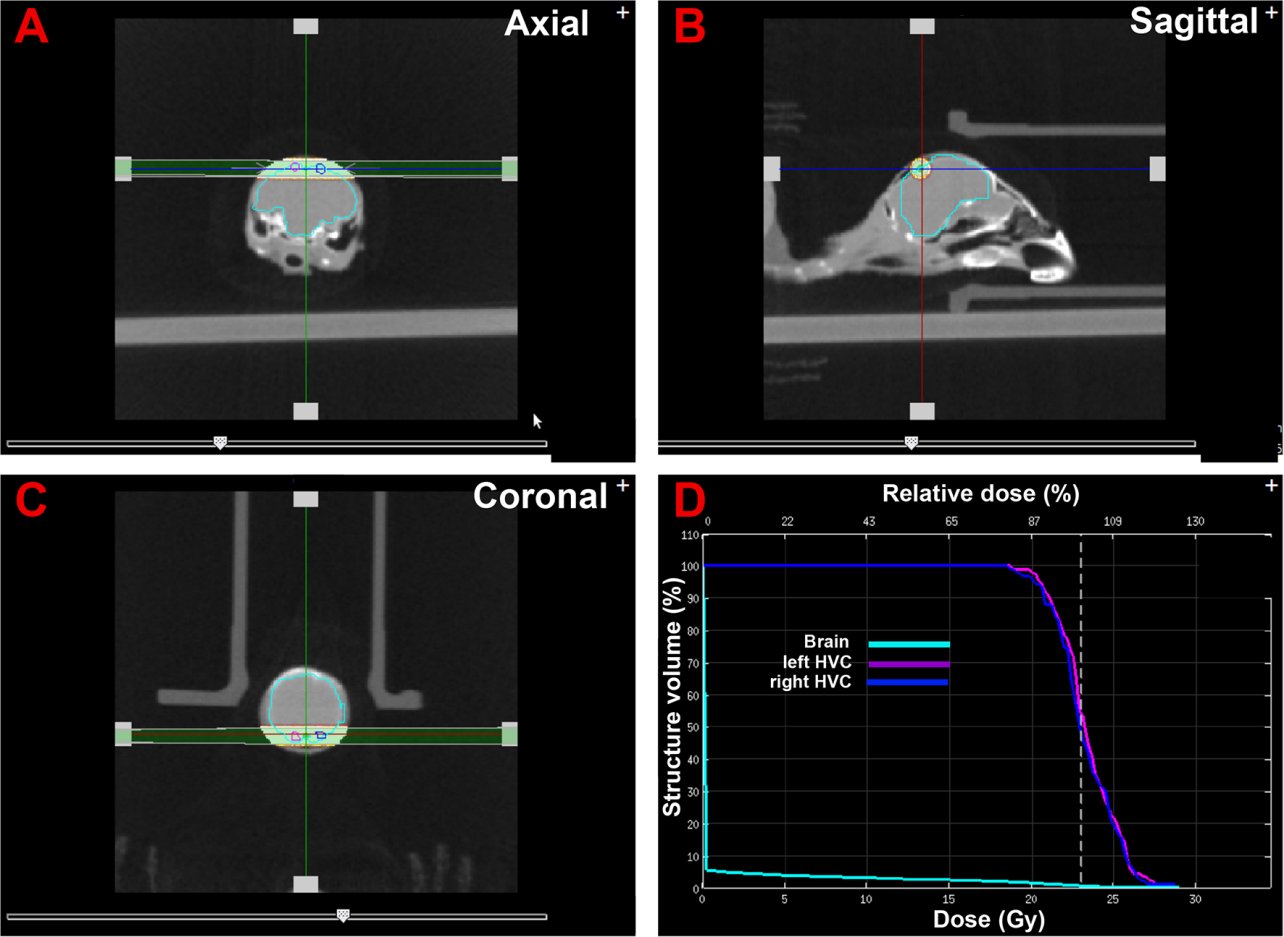
Selected copies of panels of the irradiator computer screen showing the localization of the two X-ray beams bilaterally focused on the ventricle dorsal to the 2 HVCs. (**A–C**) Illustrate the focalization of these beams in the axial, sagittal and coronal planes. (**D**) Illustrates the distribution of the energy in the left HVC (pink), the right HVC (dark blue) and in the rest of the brain (bright blue). The areas that are concerned are circled in (**A–C**) in the corresponding color. Note that the two HVCs roughly received the same energy so that the corresponding curves overlap in (**D**). About 90% of the X-ray dose was actually administered to the targeted HVCs (**D**). The 2 beams delivered the same dose of irradiation, 11.5 Gy (total 23.0 Gy) distributed over a period of 373 or 390 s for beams 1 and 2 respectively.

In experiment 2, the ventricle dorsal to HVC was irradiated in both sides of the brain with the same X-ray dosage (23 Gy). This was achieved by two horizontal beams lasting approximately 6 min coming from the left and right side of the brain (Fig. 3). Half the birds (n = 7) were assigned to an irradiation group while the other half (n = 7) was assigned to a control group that was exposed in parallel to all manipulations except for the irradiation. Each control bird was anesthetized at the same time as a matched irradiation subject and kept under anesthesia outside of the irradiator for the whole duration of the procedure on the irradiated bird.

The entire procedure lasted approximately 10–15 min per bird including the scan and irradiation. Birds in both experiments were then placed in an empty cage with a heating lamp until they were observed to perch and resume normal feeding and drinking activities, which always took place within 10 min.

### Testosterone implants

Previous studies have shown that subcutaneous Silastic™ implants filled with testosterone (Sigma, Catalog number 86500) reliably increase circulating levels of testosterone. This induces abundant singing in female canaries and increases singing motivation and induces breeding typical long-trills in males^37–39^. All subjects in experiment 1 and 2 were thus implanted with testosterone to increase the chances that birds would display an active pattern of singing behavior. Males in experiment 1 were implanted during the day preceding the irradiation procedure under isofluorane anesthesia. Females in experiment 2 were implanted immediately following irradiation or the control procedure while they were still under anesthesia.

Silastic™ implants (Dow corning reference no. 508–400, inner diameter 0.76 mm, outer diameter 1.65 mm) were 10 mm long and filled with crystalline testosterone (Fluka Analytical, Sigma-Aldrich). They were sealed with silicone and incubated in 0.9% NaCl at 37 °C overnight before implantation. A small (< 2 mm) incision in the skin was made at the base of the neck on the back of the bird, the implant was inserted down under the skin and the incision site was closed by 2 surgical sutures to prevent the implant from slipping out. Birds were monitored following the surgery during the afternoon and on the next day to ensure they were feeding normally and showed no discomfort or pain related to the implant.

### BrdU injections

To determine the effectiveness of the irradiation treatment at reducing the number of new neurons migrating and incorporating into HVC, on the day following irradiation, each subject received five intra-muscular injections of bromodeoxyuridine [BrdU, Fluka (Sigma Aldrich)] at a dose of 50 mg/kg per injection. Injections started at 8 am immediately after lights went on and continued every 2 h except for the last injection in experiment 2 which started at 3:30 pm (1.5 h interval) to allow time before lights were turned off at 4 pm. BrdU was dissolved in 0.1 M phosphate-buffered saline, PBS with 100 mM NaCl.

### Song recording and analysis

Song was recorded for 2 h per day starting at 0800 h in the morning before and after the irradiation during experiment 2. Recordings were obtained on one day immediately before the treatment and then 3 days per week for 4 weeks, starting on day 4 after irradiation and implant surgery.

Song recordings were obtained from the sound-attenuated boxes through an Allen and Heath ICE-16 multichannel recorder connected to a computer. Sound files were 16-bit sampled at a frequency of 44,100 Hz. Sound files were stored as .wav files using Raven Pro v1.4 software (Bioacoustics Research Program 2011; Raven Pro: Interactive Sound Analysis Software, Version 1.4, Ithaca, NY: the Cornell Lab of Ornithology).

Sound analysis was performed with a customized MATLAB script (program written by Ed Smith with the supervision of Dr. Robert Dooling, University of Maryland) developed specifically to detect canary songs and syllables in recordings. All recordings were analyzed channel-by-channel and day-by-day by the automated software. The software quantified multiple dependent variables for each detected song. A song was defined as a vocalization at an intensity of at least 30 dB above background that had a duration of at least 1 s and was separated from other vocalizations by at least 0.4 s.

After identifying all songs in recordings, several variables were quantified including song rate, percentage of time singing within songs, song duration, power, entropy, bandwidth, number of syllables, syllable duration and syllable power (See^41,42^ for detail and validation of the program). Measures of entropy (also called tonality coefficient) reported here are the natural logarithm (base e) of the Wiener entropy that ranges from Ln 1 = 0 for white noise to Ln 1 = minus infinity for a pure tone. This measure has been used in birdsong research namely to assess similarity of zebra finches (*Taeniopygia* *guttata*) song to the song of their tutor. In this species, birds repeat the same song over and over again and the analysis is usually focused on a small time window so that song is analyzed syllable by syllable^43,44^. In canaries, song is more variable along several dimensions including duration, number of syllables per song, syllable repertoire, sequence and frequency bandwidth. The Wiener entropy integrates all these aspects and we previously reported that the poorly structured songs of castrated males and of testosterone-treated females have larger negative values than songs of testosterone-treated males^40^.

Two measures assessed singing motivation: the song rate calculated as the total number of songs detected during the entire recording and expressed as songs/h and the mean duration of these songs. All measures collected for the three days of each week were averaged per week for the final analyses.

### Brain collection and immunohistochemistry

Birds were anesthetized with Nembutal™ (0.04 ml at 0.6 mg/ml of pentobarbital molecule). Once reflexes stopped, birds were intracardially perfused with phosphate-buffered solution (PBS), immediately followed by 4% paraformaldehyde PBS (PFA) to fix the brain. The brain was then extracted and post-fixed in PFA for 24 h and then transferred to 30% sucrose solution until it sank to the bottom of the vial, usually 24–48 h later, when it was flash-frozen on dry ice and stored at −80 °C. The two brain hemispheres were separated and cut sagittally on a cryostat into 30 μM thick sections collected into four series of two wells containing approximately 12 sections each. Sections were stored at −20 °C in anti-freeze (0.01 M PBS with 10 g/L polyvinylpyrrolidone, 300 g/L sucrose, and 300 ml/L ethylene glycol) until further use.

In experiment 2, 4 weeks after the irradiation, females were exposed to male breeding song playback for 90 min (one 30 min track repeated 3 times) starting at 0900 h. Playback was simultaneously started for two subjects (one control and one irradiated) at the same time, and playback for subsequent pairs of birds was started at 30 min intervals to allow brain tissue processing. After the 90 min of playback exposure, birds were rapidly euthanized by decapitation and their brain was extracted and fixed in a 5% acrolein solution for 2.5 h, rinsed twice in 0.1 M PBS at pH 7.4 for 30 min, after which it was placed in 30% sucrose solution at 4 °C until it sank to the bottom of the vial. The brains were then flash frozen on dry ice and stored at −80 °C. Brains were sectioned on a cryostat at 30 μM thickness in the coronal plane and sections were collected into four series, each series consisting of four wells containing approximately 12 sections each. The right hemisphere was marked before sectioning by puncturing a hole in the tissue with a small needle to allow later identification of laterality in individual sections. Sections were stored at −20 °C in anti-freeze until use.

### BrdU and doublecortin (DCX) immunohistochemistry

To quantify the effects of irradiation on the numbers of new neurons migrating and incorporating into HVC and surrounding areas, one series of sections from experiment 1 and half a series of sections from experiment 2 were co-stained for BrdU and DCX. Another half series from experiment 2 was also stained separately for DCX only. Briefly, for BrdU staining, sections were rinsed 3 × 5 min in Tris-buffered saline (TBS) to remove anti-freeze and then following every step except the saturation step (with normal donkey serum, NDS). Sections were then immersed for 20 min in 0.06% H_2_O_2_ solution in TBS, followed by a 20 min denaturing step with 2N HCl at 37 °C and a 10 min neutralization step with 0.1 M sodium borate buffer at pH 8.5 at room temperature. Sections were next immersed in blocking solution containing 10% NDS in TBS with 0.1% Triton X-100 (TBST) for 30 min followed by 24 h incubation with primary antibody (rat anti-BrdU, ABD Serotec OBT0030 lot #0410 for experiment 1 and mouse anti-BrdU Millipore MAB4072 lot 3754802 for experiment 2, each at a dilution of 1:2000) in TBST. On the following day, sections were incubated for 2 h at room temperature in secondary antibody [biotinylated donkey anti-rat for experiment 1 (Jackson Immuno Research lot# 124180) and anti-mouse for experiment 2 (Vector BA-2000 lot S0721), at 1:2000 dilution], and the signal was amplified by incubation in ABC solution (1:400 dilution of both A and B components, Vectastain Elite PK-6100, Vector Laboratories) for 90 min. The BrdU antibody binding sites were revealed using 0.04% 3,3′- diaminobenzidine (DAB), 0.012% H_2_O_2_, 0.026% ammonium nickel sulfate solution in TBS for 10.5 min.

For DCX labeling, sections were rinsed 3 × 5 min in TBS and following every step except the saturation step with normal goat serum (NGS). Sections were then immersed for 15 min in 3% H_2_O_2_ solution in TBS, 30 min in blocking solution containing 5% NGS and 1% bovine serum albumin (BSA) in TBS with 0.1% Triton X-100 (TBST). Sections were next incubated in primary antibody (rabbit anti-DCX, Abcam ab18723, lot # GR3414309-1) at a concentration of 1:5000 in TBST 5% NGS, 1% BSA for 2 h at room temperature and then 48 h at 4 °C on a rotating shaker. This was followed by incubation for 2 h in secondary antibody (biotinylated goat anti-rabbit antibody, Jackson Immuno Research lot# 145770, at a dilution of 1:500 in TBST, 5% NGS, 1% BSA) on a rotating shaker at room temperature. Sections were then incubated in the avidin–biotin complex (1:400 each A and B solution, ABC Vector Elite kit, Vector Laboratories) for 90 min and then visualized using the SG substrate kit for peroxidase (Vector laboratories, SK-4700) in a 5 min reaction. Sections were mounted on microscope slides, dried, and cover-slipped with Eukitt mounting medium (Sigma).

### Quantification of new neurons

Neurons labeled for BrdU and/or DCX were counted in photomicrographs taken with the 10X objective (Olympus BH-2 microscope) with a color digital camera (Scion Corporation CFW-1612C). Fusiform DCX-positive cells (youngest cells still in migration) and multipolar DCX-positive cells (older cells that have begun their final differentiation, see^45,46^) were separately counted.

In experiment 1, neurogenesis was quantified in all sections containing HVC (3 sections for 6 males and 4 sections for 2 males). We counted separately BrdU-positive (BrdU^+^) cells that were also expressing DCX (DCX^+^ fusiform or multipolar) or were not expressing this marker of new neurons. We also quantified the total number of fusiform or multipolar DCX^+^ new neurons. The area of these same HVC sections was then measured with the help of the ImageJ 1.53a software (Wayne Rasband, National Institutes of Health, Bethesda MD) in photomicrographs taken at 4× of this nucleus. The software was first calibrated with a picture of a known-size ruler and the perimeter of the nucleus was then delineated with the computer mouse using the free-form tool. The software calculated the area of the nucleus in the section that was used to calculate for each section the density of each cell type (number divided by area in mm^2^). Statistical analyses then considered the mean density (number/mm^2^) of each type of cells in the 3–4 sections on the irradiated and control non-irradiated hemisphere.

The same procedure was used for experiment 2, but since immunostaining for DCX was, for unidentified reasons, of poor quality in a small number of double-labeled sections, DCX counts were additionally collected in one section that had been labeled for DCX only. All measures of densities were then averaged for all sections (1 to 8 per bird) where they had been quantified and used for statistical analyses.

### ZENK immunohistochemistry

In experiment 2, during which females were exposed to 90 min of male song playback before brain collection, half a series of brain sections was stained by immunohistochemistry to visualize the ZENK protein (an acronym widely used in birds for zif-268, egr-1, NGFI-A and Krox-24, the corresponding genes cloned in mammals)^47^. Briefly, sections were rinsed 3 × 5 min in TBS to remove anti-freeze and then following every step except the saturation step (with goat serum, NGS). Sections were immersed for 20 min in 0.06% H_2_O_2_ solution in TBS, followed by blocking solution containing 5% NGS in 0.1% TBST for 30 min followed by 24 h incubation with primary antibody (rabbit anti-egr-1 at a concentration of 1:2000, Santa Cruz SC-189) in 5% TBST at 4 °C on a rotating shaker. The following day, sections were incubated for 2 h at room temperature in secondary antibody (biotinylated goat anti-rabbit at 1:400 dilution, Vector BA-1000 lot# K0206), and the signal was amplified by incubation in ABC solution (1:800 dilution of both A and B components, Vectastain Elite PK-6100, Vector Laboratories) for 90 min. The ZENK antibody binding sites were revealed using 0.04% 3,3′- diaminobenzidine (DAB), 0.012% H_2_O_2_, 0.026% ammonium nickel sulfate solution in TBS for 5 min. The specificity of the ZENK antibody used here has been previously validated specifically in canaries^48,49^.

To quantify ZENK expression, photomicrographs were taken with the 20× objective at 3 locations in the brain as defined in^50^: the caudomedial mesopallium (CMM), the rostroventral caudomedial nidopallium (rvNCM) and mediocaudal NCM (mcNCM, at the level of HVC) where these authors had identified clear ZENK induction by song. The area covered by the ZENK-immunoreactive nuclei was measured in the entire photomicrographs (370 × 275 µM) using a semi-automatic Fiji routine that involved subtracting the background color, manually adjusting the threshold, and then running the “analyze particles” function that calculated area covered by positive material. This area covered by immunoreactive material was then divided by the total area considered to provide the percentage of area (% area) covered.

This measure was validated by correlating the measured area covered to the number of positive cell nuclei in all 14 photomicrographs taken on the left and right hemispheres in CMM, rvNCM and mcNCM. This procedure demonstrated a positive correlation between area covered and number of cell nuclei but the corresponding correlation coefficient was quite variable in the range 0.542 to 0.902. We realized that this variability was presumably caused by the partial overlap between cell nuclei in the photomicrographs. Therefore in a subset of pictures (the left side of the all CMM photomicrographs), the routine was modified to count only single nuclei without overlap (by changing the range of size and circularity of acceptable particles). Then the overlapping cell nuclei were manually counted and added to the automatic count. This demonstrated an excellent positive correlation between the area covered and the total number of cell nuclei expressing ZENK counted in this composite manner (r = 0.921, n = 14, p < 0.0001). This procedure was used to generate the data set that are presented in this paper.

### Song control nuclei volume calculations

To test whether the bilateral irradiation in experiment 2 had affected the overall volume of the song control nuclei, one series of sections was Nissl-stained. Sections were mounted on Superfrost slides and dried overnight. Sections were then rehydrated and immersed in toluidine blue solution for 2.5 min followed by two Walpole buffer baths of 15 and 18 min each. Slides were immersed in a molybdate solution to fix the color, dehydrated in a series of isopropanol solutions of increasing concentrations and finally in xylene before cover-slipping.

Photomicrographs of all sections containing HVC, RA or Area X of the basal ganglia were collected in both hemispheres with an Olympus BH-2 microscope connected to a Scion Corporation CF2-1612C digital color camera. Photomicrographs were obtained with a 4X objective using the same light settings throughout. Brain nuclei volumes in these pictures were measured with ImageJ 1.53a, first by calibrating the software with a picture of a known-size ruler and then delineating the perimeter of the nucleus in the section, which resulted in an automatic measurement of the area. In a few cases when a section was damaged, the surface of the nucleus in this section was estimated as the average of the area in adjacent sections. In addition HVC volume could not be determined in one control male due to damage of the sections. The volume of the nuclei was calculated by summing the areas in all ipsilateral sections and multiplying by 0.120 mm (120 μM), the distance between two successive sections in the series.

### Statistical analyses

Data were analyzed by Student t-tests or 2/3-way analyses of variance (ANOVA), eventually with a factor considered as repeated measure, as appropriate for the design of data. If a few individual points were missing for technical reasons, the ANOVA was replaced by the corresponding mixed-effects analysis. Missing points were namely present in the analysis of the singing behavior. If a bird did not sing on a given day, it was assigned a singing rate and singing duration of zero but characteristics of the song structure could obviously not be determined and these missing values automatically induced a switch to a mixed-effect analysis. This small number of missing data points will not be discussed in a case by case but their number can always be deduced from the degrees of freedom of all analyses. All analyses were performed with Prism 8.4 for macOS (GraphPad Software LLC, San Diego CA). Effects were considered statistically significant for p < 0.05. All data are represented by their mean and SEM but individual data points are additionally presented in the graphs in most cases.

## Results

### Experiment 1

#### Neurogenesis

**Figure 4:**
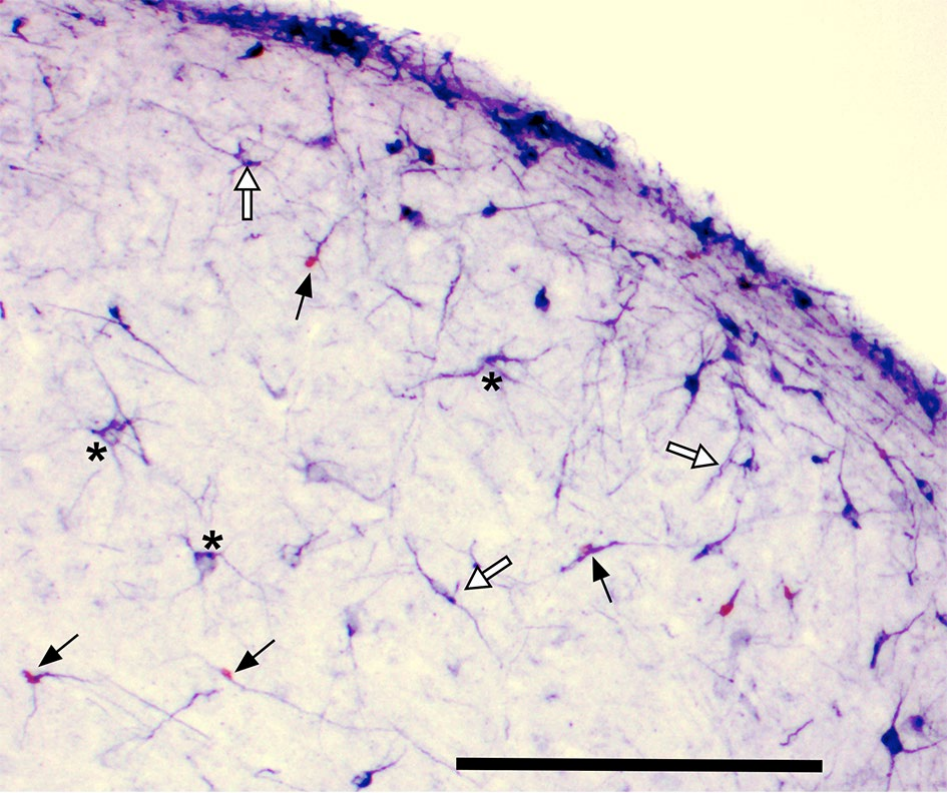
Photomicrograph of a section from a control bird through HVC stained by immunocytochemistry for BrdU (brown) and DCX (dark blue). Examples of nuclear BrdU located in fusiform DCX+ neurons are indicated by arrows, Examples of multipolar DCX positive cells showing a clear nucleus are indicated by an asterisk. Fusiform DCX+ cells without BrdU are indicated by open arrows. Magnification bar = 200 µM.

The immunohistochemically stained sections clearly identified BrdU-positive cells and DCX-positive neurons. As reported previously^45,46,51^, the BrdU label was exclusively nuclear while DCX stained the entire cytoplasm including all cellular processes but leaving a clear nucleus. BrdU was essentially located in relatively young neurons displaying a fusiform morphology. (Fig. 4).

**Figure 5:**
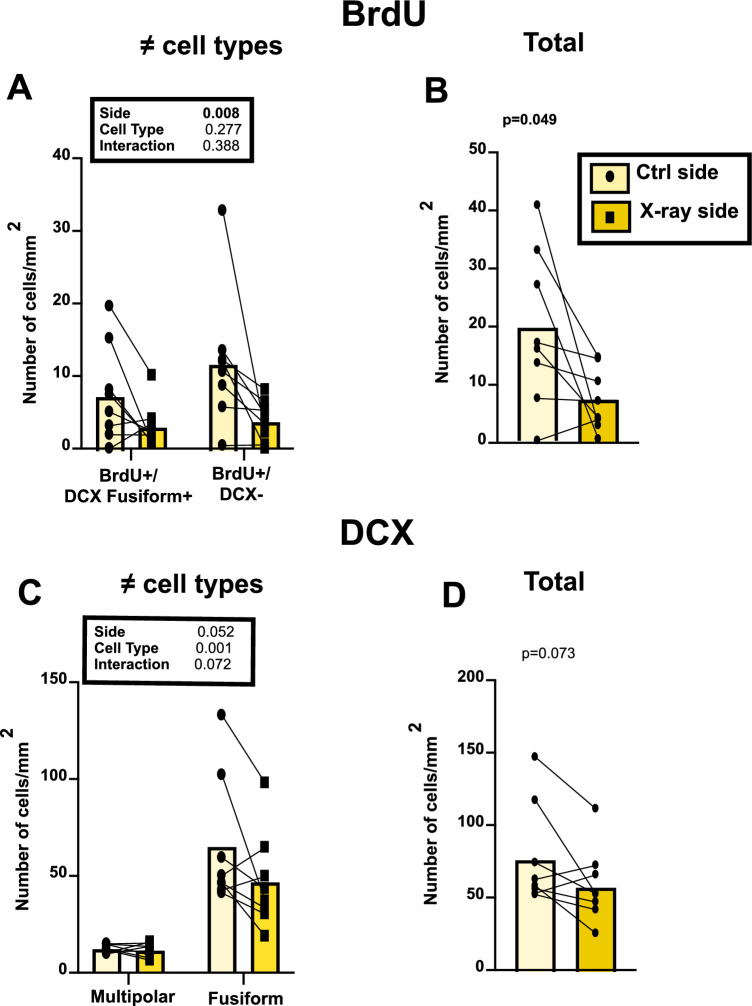
Quantification of neurogenesis in the irradiated (X-ray) and control (Ctrl) HVC of male canaries as assessed by BrdU incorporation and by the expression of DCX in young neurons. Densities (numbers/mm^2^) of two different cell types were analyzed by two-way ANOVAs and results are summarized in the corresponding inserts. Total densities were analyzed by paired Student t-tests and corresponding probabilities are indicated at the top of the graphs.

The quantification of neurogenesis by the measure of BrdU incorporation and of the expression of DCX provided converging evidence for a clear effect of the focal X-ray irradiation. Close inspection of all sections failed to identify a single multipolar DCX^+^ cell that also contained BrdU^+^. This was anticipated since brains were collected two weeks after the BrdU injections and new neurons first display a fusiform morphology and only begin their final differentiation and acquire a multipolar morphology after at least one month^45,46,51^. BrdU was in contrast observed in DCX^+^-fusiform neurons and in cells that were DCX negative and these numbers were decreased by more than 50% in the irradiated side (Fig. 5A). Analysis of the density of these two cell types by a mixed-model two way ANOVA (2 cell types as independent factor and 2 brain sides as repeated factor) identified a significant effect of the brain side (i.e., the irradiated vs. control hemisphere; F_1,14_ = 9.562; p = 0.008) but no overall effect of the cell type (F_1,14_ = 1.279; p = 0.277) and no interaction between these factors (F_1,14_ = 0.791; p = 0.389). The cumulative density of these two cell types was also significantly different between the irradiated and the control side (7.36 ± 1.89 vs. 19.54 ± 4.74; paired Student t_7_ = 2.384, p = 0.049; Fig. 5B).

Since DCX is expressed in all neurons during the first one or two months of their life^45^, whereas BrdU only labels new cells during a maximum of two hours after its injection^52^, HVC contained more neurons expressing DCX than cells containing BrdU. In addition, as explained before, since DCX^+^ cells only become multipolar after one month, all multipolar DCX^+^ cells detected were actually born before the irradiation and their number was not affected. In contrast, there was a substantially lower number of DCX + -fusiform neurons on the irradiated hemisphere by comparison with the control side (Fig. 5C). Analysis of these two types of cells by two-way ANOVA accordingly indicated a nearly significant effect of brain side (F_1,14_ = 4.523; p = 0.052) and of the interaction between brain side and cell type (F_1,14_ = 3.780; p = 0.072) associated with a difference between cell types (F_1,14_ = 21.28; p < 0.001). The total density of DCX^+^ neurons similarly indicated a strong trend toward a decrease in the irradiated hemisphere (58.52 ± vs. 77.50 ± 12.47: paired Student t_7_ = 2.103, p = 0.073; Fig. 5D). There was however no overall difference between the average cross-sectional area of all HVC where DCX had been measured in the two hemispheres indicating that the depletion of new neurons was probably not sufficient to induce a change in HVC volume (Ctrl: 0.450 ± 0.024, X-ray: 0.453 ± 0.018, mean ± SEM, paired Student t_7_ = 0.060, p = 0.954).

## Experiment 2. Bilateral irradiation in females

### Neurogenesis in HVC

**Figure 6:**
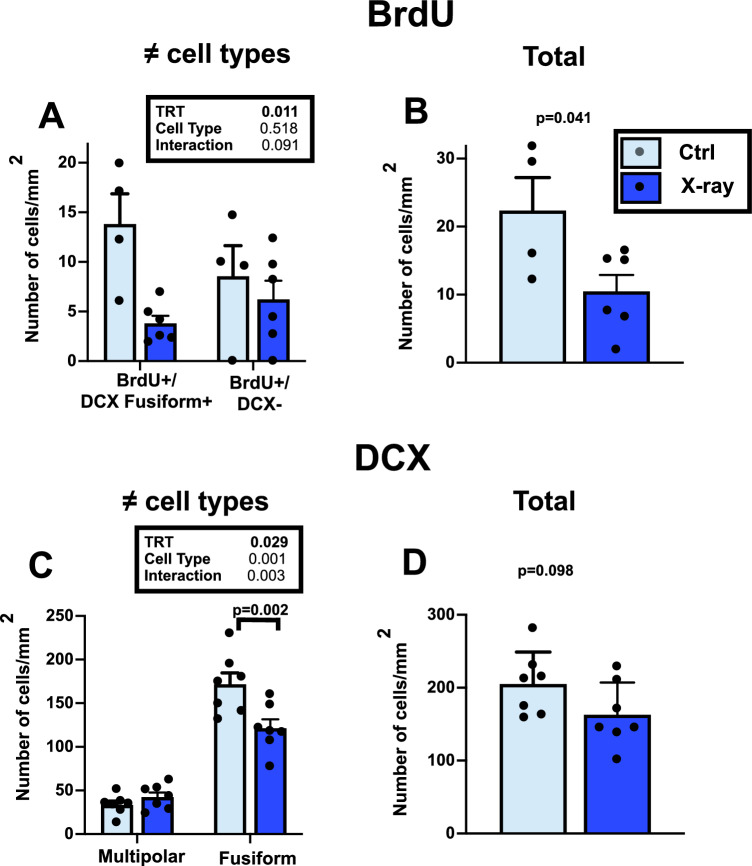
Quantification of neurogenesis in the irradiated (X-ray) and control (Ctrl) HVC of female canaries as assessed by BrdU incorporation and by the expression of DCX in young neurons. Densities (numbers/mm^2^) of two different cell types were analyzed by two-way ANOVAs and results are summarized in the corresponding inserts (*TRT* treatment, *Int* interaction). Total densities were analyzed by paired t-tests and corresponding probabilities are indicated at the top of the graphs.

As observed in experiment 1, almost no multipolar DCX^+^ neuron contained BrdU. This BrdU accumulation was actually observed in only one subject of the X-ray group and it was present in only 1 or 2 neurons per section. BrdU was essentially observed in DCX^+^-fusiform neurons and in DCX^-^ cells (Fig. 6). Analysis of these data by two-way ANOVA, however, did not reveal an overall difference in densities of the two cell types (F_1,16_ = 0.437, p = 0.518) nor an interaction between cell type and treatment (F1_,16_ = 3.234, p = 0.091) but there was an overall difference in the density of BrdU^+^ cells linked to treatment (F_1,16_ = 8.344, p = 0.011) that was lower in the HVC of irradiated females (Fig. 6A). Accordingly analysis by Student t-test of the total density of these BrdU^+^ cells indicated a lower density in irradiated HVC (10.50 ± 2.41 vs. 22.37 ± 4.85; t_8_ = 2.435, p = 0.041; Fig. 6B).

As in experiment 1, the density of DCX^+^ neurons was, in this case also, higher than the density of BrdU^+^ cells, and fusiform neurons were denser than multipolar ones. Statistical analysis of the densities of these two cell types accordingly revealed a significant difference between the two cell types (F_1,24_ = 147.4, p < 0.001) and most importantly a significant difference between densities in control and irradiated birds (F_1,24_ = 5.371, p = 0.029; Fig. 6C). There was also a significant interaction between these two factors (F_1,24_ = 10.93, p = 0.003) resulting from the fact that densities of fusiform cells were decreased in irradiated birds (p = 0.002 by Sidak’s multiple comparisons test) but no significant difference between group was present in the multipolar neurons (p = 0.741). The total number of DCX + cells did not differ between the two groups (Ctrl: 205.1 ± 16.53; X-ray: 162.9 ± 16.70; t_12_ = 1.796, p = 0.098; Fig. 6D).

**Figure 7:**
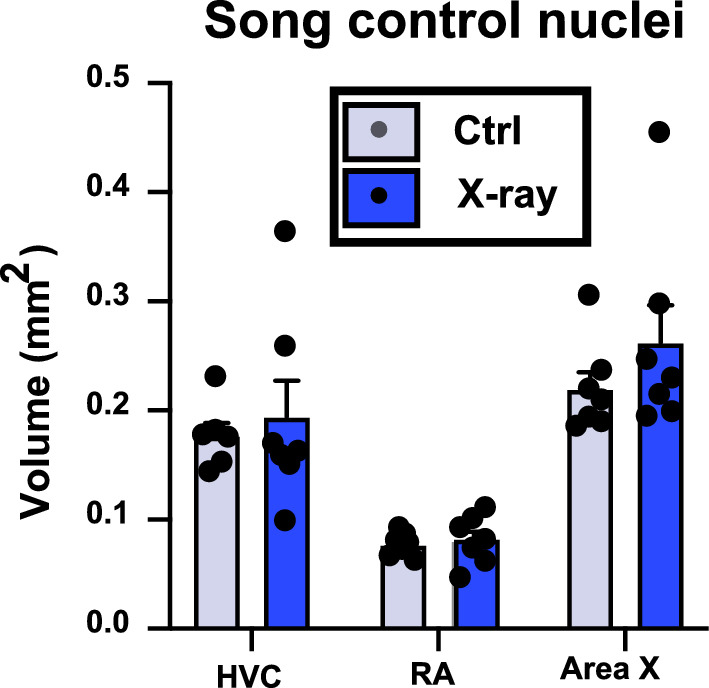
Volume of the song control nuclei in X-ray-irradiated and control testosterone-treated females.

As in experiment 1, this decreased neurogenesis in the X-rayed subjects was apparently not sufficient to decrease HVC volume as suggested by the finding that the average cross-sectional area of all HVC sections where DCX had been measured was not affected (Ctrl: 0.320 ± 0.026, X-ray: 0.364 ± 0.025 mean ± SEM, t_12_ = 1.215, p = 0.248). This conclusion was formally confirmed by measuring in Nissl-stained sections the volume of these nuclei. These data were initially analyzed by a 3-way ANOVA with the treatment, brain nucleus and brain side (repeated factor) as factors, but since no effect of brain side and no interaction of brain side with the other factors was detected (all p ≥ 0.092), this factor was removed from the analysis that was redone by a two-way ANOVA based on the means of the left and right hemisphere. This analysis (Fig. 7) confirmed the lack of overall effect of the treatment on these volumes (F_1,35_ = 1.394, p = 0.246) and the absence of interaction between treatment and nucleus type (F_2,35_ = 0.399 p = 0.674), even if the absolute volume of these 3 nuclei was obviously different (F_2,35_ = 28.610, p < 0.001). Even a t-test focused on HVC volumes only (the nucleus directly affected by X-rays) failed to find any sign of difference (t_11_ = 0.467, p = 0.651).

### Singing behavior

**Figure 8:**
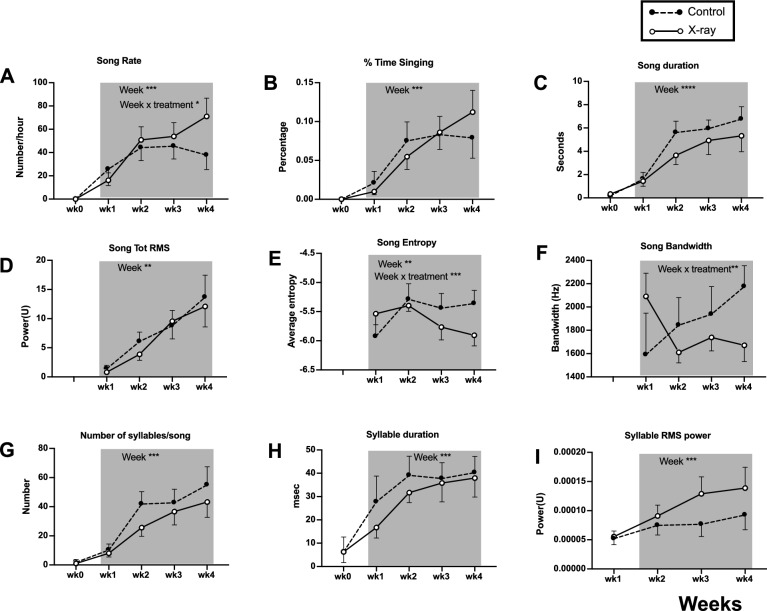
Song rate and song features of female canaries before (wk0) and after (wk1 to 4, shaded grey box) X-ray irradiation. Significant effects identified by two-way ANOVA or mixed-effects models with treatment and time as factors are indicated when significant using asterisks (***p < 0.001, **p < 0.01, *p < 0.05). See Table 1 for the detail of statistical results.

As expected, female song output was low prior to treatment with testosterone with only three females producing ≥ 1 song/h (Fig. 8). Following testosterone treatment, which is known to increase the singing motivation and fine-tune song features in both male and female canaries (e.g.^38,40^), many song variables, particularly related to song motivation and power, showed an increase over the course of the four weeks post-treatment. An overall effect of time (weeks) was actually detected for all dependent variables except song bandwidth (Fig. 8, Table 1).

**Table 1:** Results of two-way ANOVA or mixed-model analysis of female song variables.

Variable	Week	Week	Treatment		Week × treatment	
	F	p	F	p	F	p
Song rate (songs/h)	F (4,48) = 22.090	** < 0.001**	F (1,12) = 0.389	0.544	F (4,48) = 2.582	**0.049**
Proportion of time spent singing	F (4,48) = 24.090	** < 0.001**	F (1,12) = 0.002	0.961	F (4,48) = 1.382	0.254
Average song duration (s)	F (4,48) = 29.950	** < 0.001**	F (1,12) = 1.076	0.320	F (4,48) = 0.882	0.419
Song total RMS	F (3,30) = 16.400	** < 0.001**	F (1,12) = 0.102	0.755	F (3,30) = 0.312	0.817
Song average entropy	F (3,30) = 4.901	**0.007**	F (1,12) = 0.211	0.654	F (3,30) = 9.688	** < 0.001**
Song bandwidth (Hz)	F (3,30) = 1.064	0.379	F (1,12) = 0.178	0.685	F (3,30) = 6,782	**0.001**
Mean number of syllables	F (4,48) = 24.040	** < 0.001**	F (1,12) = 0.939	0.352	F (4,48) = 0.606	0.660
Syllable duration (msec)	F (4,48) = 17.520	** < 0.001**	F (1,12) = 0.303	0.592	F (4,48) = 0.474	0.754
Syllable RMS power	F (3,30) = 7.328	** < 0.001**	F (1,12) = 0.877	0.367	F (3,30) 1.291	0.295

Significant values are in bold.

No overall effect of the treatment was detected but an interaction between weeks and treatment was observed in three cases. This interaction concerned the song rate, song entropy and song bandwidth. Post-hoc Sidak’s tests comparing controls to X-rayed birds on each separate week failed however to identify any significant treatment difference.

**Figure 9:**
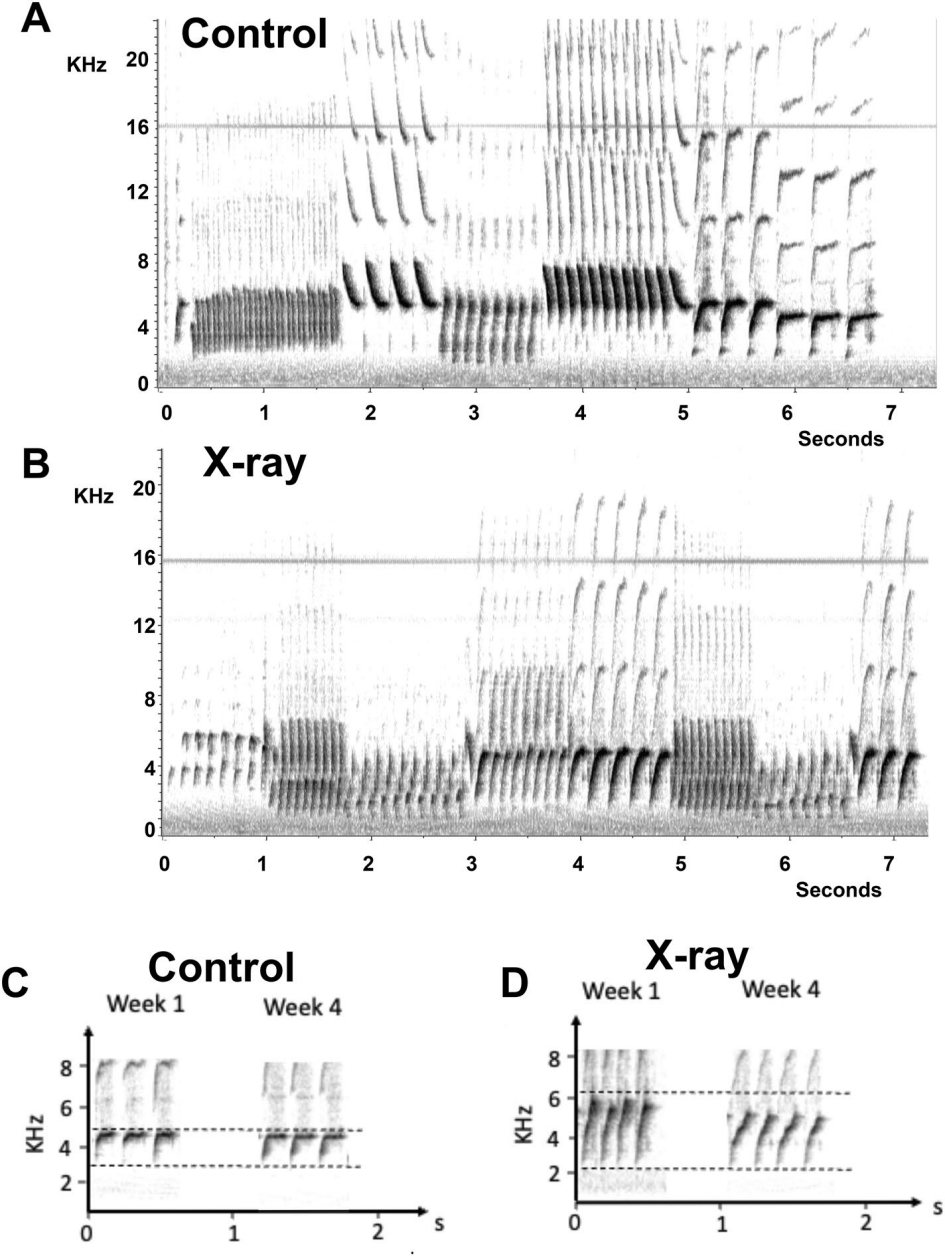
Example of sonograms of a song produced by a control (**A**) and an X-ray female (**B**) and of a syllable that was progressively sung with a decreased bandwidth after the irradiation (**D**) but not in a control bird (**C**). Comparisons are presented between week 1 and 4 because females did not sing before the implantation of testosterone and comparisons with week 0 (pre-test) cannot be made.

Surprisingly, the overall song rate increased more in irradiated birds than in controls but in contrast, song entropy and song bandwidth decreased after irradiation as compared to controls. The more negative values of the logarithm of the Wiener entropy (smaller values of entropy itself) were associated with a decrease in bandwidth, which could, in part at least, explain the change in entropy. Other aspects of song that were not quantified here could of course also contribute to the decrease in entropy. As illustrated in Fig. 9A, B, there was no gross modification of song after irradiation but more subtle differences could obviously be present. Note also that there was a non significant trend in irradiated birds to include fewer syllables in each song and this could also contribute to the lower entropy value of their songs. These songs of irradiated birds were thus more similar to the songs of castrated subjects^40^ (Fig. 9).

Additional analyses might be needed to more precisely identify the nature of this effect but visual inspection of the sonograms clearly confirmed that many syllables in irradiated females had a narrower bandwidth compared to the control subjects. This decreased bandwidth is difficult to observe in full sonograms because different females do not sing the same repertoire of syllables but it is best documented in the comparison of a same syllable recorded in the same bird at the very beginning (week 1) and at the end (week 4) of the experiment as illustrated in Fig. 9C, D: a decrease is seen in the X-rayed female but not in the control bird.

### Immediate early gene expression in female auditory brain areas

Females were exposed to male song playback for 90 min before brain collection and ZENK expression was quantified in three auditory areas known to respond to variation in song quality (e.g.^50,53^) with the help of a semi-automatic protocol in ImageJ. Once again, these data were initially analyzed by a three-way ANOVA (treatment, brain area and hemisphere side) but no lateralization and no interaction between lateralization and the other factors was detected (all p ≥ 0.149) so this factor could be removed from the analysis.

**Figure 10:**
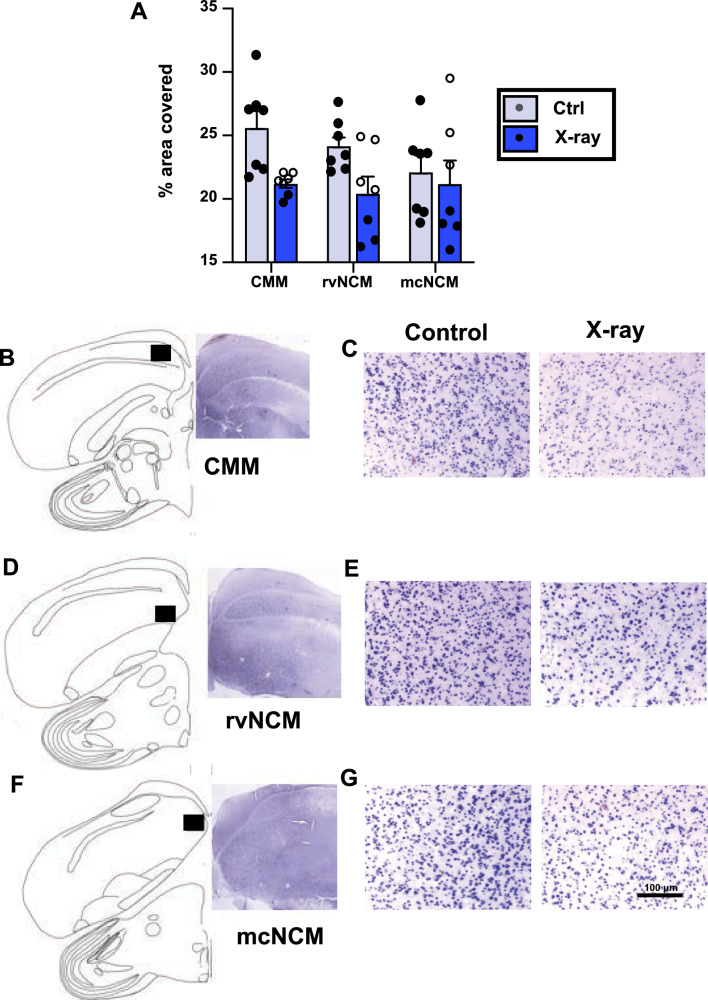
Effect of X-ray irradiation on ZENK expression in three auditory areas, the caudomedial mesopallium (CMM), the rostroventral caudomedial nidopallium (rvNCM) and the mediocaudal NCM (mcNCM, at the level of HVC). (**A**) Summarizes the quantitative data, (**B,D,F**) present schematic drawings of the areas that were investigated (black rectangles) as well as photomicrographs of the corresponding regions in sections stained by immunohistochemistry for ZENK. (**C,E,G**) Contain examples of high magnification photomicrographs of the quantified areas in control (left) and irradiated (right) areas in sections stained by immunohistochemistry for ZENK. The magnification bar in the lower right panel (100 µM) applies to all high magnification photomicrographs in (**C,E,G**).

Analysis of the average data for the two hemispheres by two-way ANOVA (treatment and brain areas as factors) identified an overall effect of treatment (F_1,36_ = 8.790, p = 0.005), but there was no difference between areas (F_2,36_ = 1.009, p = 0.374) and no interaction between areas and treatment (F2,36 = 1.104, p = 0.343) (Fig. 10).

Although no significant interaction between treatment and area was detected, the qualitative observation of data in Fig. 10A suggested that the effect of irradiation was most pronounced in CMM. Accordingly, separate t tests corrected for multiple testing indicated the presence of a significant difference in CMM (t_12_ = 3.212, p = 0.022), a statistical trend in rvNCM (t_12_ = 2.432, p = 0.062) and absolutely no difference in mcNCM (t_12_ = 0.405, p = 0.693).

The observed difference in ZENK expression could be the result of differential activation by the auditory stimuli but also of a difference in cell densities in the target areas. To test the second of these possibilities, the total number of cells, irrespective of their phenotype, was counted in the Nissl-stained sections in the two auditory areas showing treatment effects or trends of effects. Comparisons by t tests revealed no difference in cell density between treatment groups in these regions (CMM: Ctrl = 677 ± 11, X-ray = 686 ± 19, t_12_ = 0.492, p = 0.682; rvNCM: Ctrl = 666 ± 18, X-ray = 684 ± 29, t_12_ = 0.548, p = 0.59).

**Figure 11:**
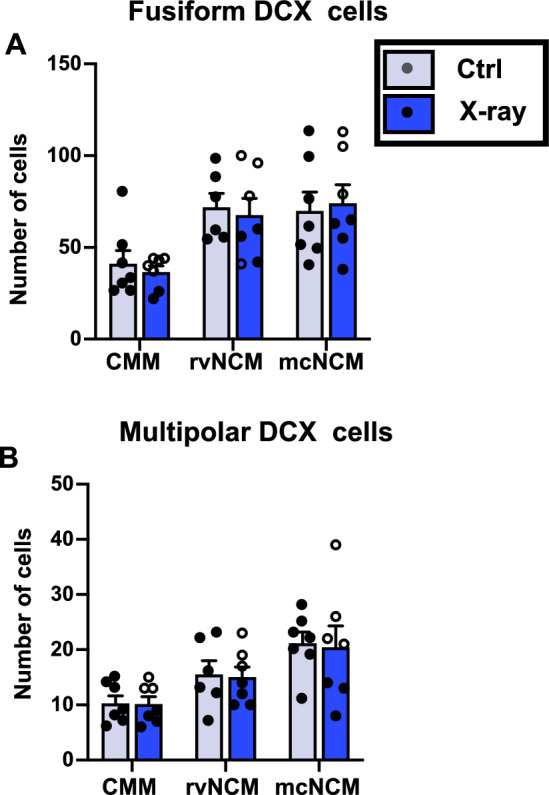
Numbers of fusiform (**A**) and multipolar (**B**) DCX^+^ cells in the irradiated (X-ray) and control (Ctrl) auditory areas of female canaries where differences in ZENK induction after song play back have been observed.

Because these auditory areas are relatively close to the irradiated area, a difference in new neuron incorporation too small to be reflected in the total number of cells remained a possible explanation for the decreased ZENK induction. Therefore DCX^+^ cells were counted on both sides of the brain in CMM, rvNCM, and mcNCM, in the same areas where ZENK expression in response to male breeding song had been quantified. These analyses identified regional differences in the density of both types of DCX^+^ cells (fusiform: F_2,35_ = 9.860 p < 0.001; multipolar: F_2,35_ = 10.620, p < 0.0001) but there was no difference associated with the irradiation treatment (fusiform: F_1,35_ = 0.048, p = 0.827; multipolar: F_1,35_ = 0.057, p = 0.813) and no interaction between treatment and area (fusiform: F_2,35_ = 0.172, p = 0.842; multipolar: F_2,35_ = 0.008, p = 0.992; Fig. 11).

### Correlation between the neurogenesis inhibition and physiological effects

**Figure 12:**
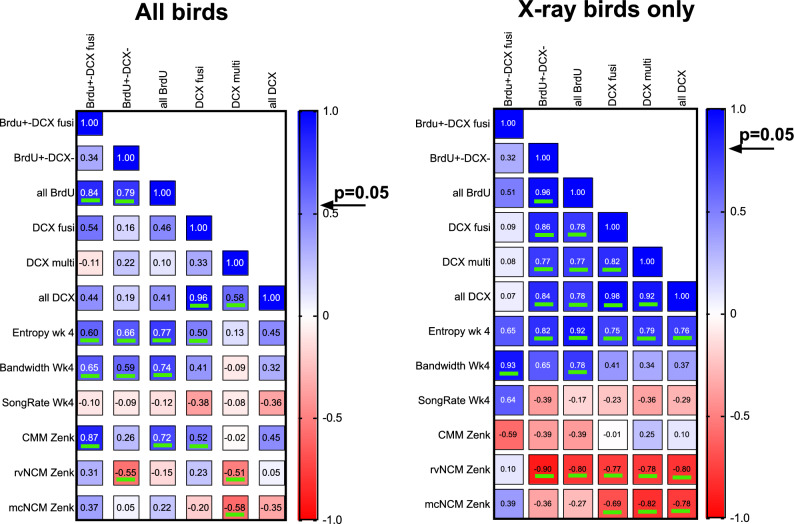
Heatmaps of correlation coefficients relating measures of neurogenesis in HVC to the behavioral and physiological variables that were significantly affected by the irradiation. The left side shows the correlation coefficients calculated for all birds considered (irradiated and their controls) together while the right- side concerns coefficients calculated for the irradiated birds only. The color legend on the right indicates the color code used to highlight the correlation coefficients. Arrows next to the color legend indicates the lowest one-tailed correlation level significant at p = 0.05. These significant correlations (p < 0.05) are underlined with a thick green line.

Finally, we asked whether the magnitude of the behavioral and physiological effects detected were related at the individual level to the degree of inhibition of neurogenesis that had been induced. Correlations were therefore calculated between all brain measures related to neurogenesis and the changes in singing and ZENK expression in auditory areas that had been found significant (Fig. 12).

As can be seen in Fig. 12 left panel, the different measures of neurogenesis based on BrdU incorporation and DCX neurons density were in general positively correlated (coded in blue in the figure). In addition, the three variables significantly affected by the irradiation treatment (entropy and bandwidth measured on week 4, ZENK density in CMM), were positively associated with measures of neurogenesis (only blue squares) and correlations with the number of BrdU^+^-DCX^+^ fusiform cells as well as with the total number of BrdU^+^cells were statistically significant (p < 0.05). In contrast, the DCX^+^ multipolar cells were not significantly correlated with these three behavioral or physiological variables.

These correlations had initially been calculated for both groups of birds (control and irradiated subjects) together (Fig. 12 left). Because these correlations could reflect the group differences only, correlations were also computed separately for the group of irradiated birds. The same pattern of positive correlations essentially emerged except that the relationship with ZENK expression in CMM now disappeared: these variables were now linked by negative correlations although these correlation coefficients were far from significant. Also, due to the limited number of data points and thus decreased power of the analyses a few of these correlations became statistically non-significant (see detail in Fig. 12 right side).

**Figure 13:**
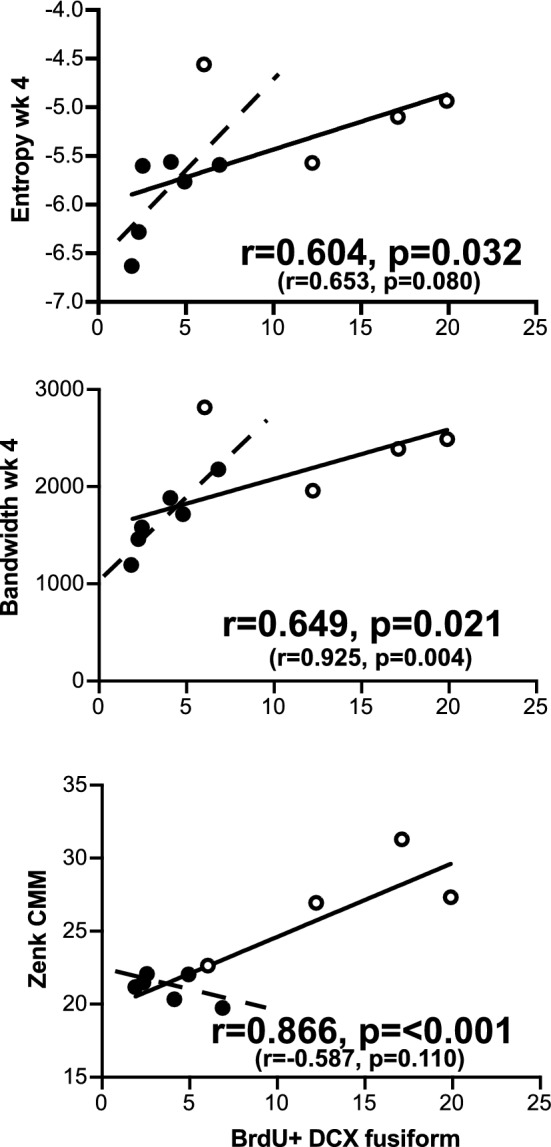
Linear regressions relating one measure of neurogenesis in HVC (the density of BrdU^+^-DCX^+^ fusiform neurons) to the behavioral and physiological variables that were significantly affected by the irradiation. Irradiated birds are represented by black data points and controls by open data points. The full lines represent the regressions based on all birds (correlation coefficient and associated one tailed probabilities in large font) while the dotted regression lines and correlations and probabilities in smaller font and in parentheses refer to the analyses of irradiated birds only.

The detailed analysis by linear regression of the relationships between BrdU^+^-DCX fusiform neurons and the three variables affected by HVC irradiation (Fig. 13) confirmed the lower overall rate of adult neurogenesis in the HVC of irradiated birds (black data points gathered on the left side of the graphs indicating smaller density of BrdU^+^-DCX fusiform neurons). This analysis also showed that an inhibited neurogenesis was associated at the individual level with lower values of entropy (more variable songs) and a lower bandwidth but the relationship with ZENK in CMM was based on the group differences only.

## Discussion

We demonstrated that X-ray irradiation substantially depletes the recruitment of young neurons in the HVC of male and female canaries. This inhibition of neurogenesis was accompanied in females by an unexpected increase in testosterone-induced song rate and by specific changes in song quality. There was also a significant decrease in the activation of secondary auditory areas of the telencephalon in response to male song as measured by the induction of ZENK immediate early gene expression. These observations raise multiple questions.

### Technical aspects

A statistically significant depletion of HVC neurogenesis was observed in both experiments. When measured by BrdU incorporation, this depletion reached 50% or more, indicating that during the day that followed irradiation very few new neurons were produced. BrdU indeed only remains available in the circulation for incorporation for about 2 h after its injection^15^. The five injections could thus only label cells replicating their DNA during the day following irradiation.

In contrast, DCX labels all new neurons for one month or more^51^. Accordingly, many more neurons were DCX^+^ compared to neurons labeled with BrdU. Depletion measured by DCX^+^ neurons density was also more limited (20–25%). Either a substantial fraction of these cells was already born before irradiation or the inhibition of neurogenesis by X-rays was only temporary and neurogenesis progressively resumed. More extensive time-course studies would be needed to discriminate between these possibilities. The lack of depletion of presumably older DCX^+^-multipolar cells, suggests that, as expected, the X-rays did not affect the neurons that were already born at the time of irradiation. Note also that BrdU labels all cells replicating DNA, whereas DCX is a specific marker of new neurons. The fact that in both experiments some BrdU^+^ cells were negative for DCX and that there was a decrease in this type of cells after irradiation thus suggests that, as anticipated, the X-rays ablated progenitor cells irrespective of whether they will later acquire a neuronal or a glial phenotype. The relative magnitude of the two cell types (glial vs. neuronal) in the BrdU^+^-DCX^-^ population cannot be determined based on the present data and similarly, whether depletion of non-neuronal cells contributed to the functional effects observed remains an unanswered question.

Based on these quantitative analyses, it appears that the irradiation procedure used here quite effectively depletes neurogenesis in HVC. This depletion is however not complete, as is the case for all other procedures used to manipulate neurogenesis. An obvious reason is that the dose of 23 Gy might be too low, but increasing it would possibly generate non-specific detrimental effects. Alternatively, the new neurons recruited in HVC are possibly not originating only from the ventricular wall adjacent and dorsal to the nucleus as suggested by several studies^54,55^; a contribution of cells from other sites of origin cannot be excluded. Some neurons recruited in HVC might originate from the hot spot of neurogenesis located at the ventral tip of the lateral ventricles^15,56^. This neurogenic niche should not have been affected by the irradiation procedure since it was focused to the most dorsal part of the brain. Finally, it is possible that irradiation decreases only temporarily the process of neurogenesis and some recovery takes place in the following weeks. It is impossible to discriminate among these ideas at present. But it is critical to note that the depletion of neurogenesis by irradiation was clearly sufficient to induce functional deficits in song production and perception.

### Effects on singing behavior

In experiment 2, treatment with exogenous testosterone induced the development of relatively complex songs in both the control and the irradiated females. Many features of these songs were similar in the two groups, suggesting that the irradiation procedure did not simply lesion HVC; if this were the case, singing would simply be inhibited. Detailed analyses identified however three statistically significant interactions between time and the X-ray treatment.

The increased song rate in irradiated birds might seem a priori counter-intuitive but it might be reminiscent of the very high song rate observed during the period of plastic song in the ontogeny of this species^57^. Since these birds were producing mostly imprecise and variable songs, it might be speculated that they were practicing more frequently in an attempt to correct by trial and errors as observed for song practice during plastic song in ontogeny^57^.

The more negative song entropy and decreased song bandwidth are more interesting. More negative values of the Wiener entropy might reflect, at least in part, the fact that energy of songs in irradiated females was distributed in a narrower bandwidth than in controls, but it is clear that other aspects of the songs could also contribute and should be investigated. These more negative entropy values and narrower bandwidth of songs after inhibition of neurogenesis fit in well with what is known of the role of HVC in the control of song. It was indeed shown that implantation of the antiandrogen flutamide in HVC increases the variability of syllable usage by male canaries as well as the bandwidth variability^58^. The depletion of new neurons might thus have similar effects as the blockade of androgen action. Androgen or its metabolites are facilitating the incorporation of new neurons in HVC^14^ and thus the effects of blocking androgen action in HVC or directly inhibiting neurogenesis are similar. This similarity might however be only superficial since androgens, in addition to their action on the incorporation of new neurons, also have other effects such as increasing neuronal size and the size of the dendritic arborization of neurons. This could only be ascertained by detailed studied tracing the arborization of existing and newly incorporated neurons.

New HVC neurons added in adulthood are mostly if not exclusively RA-projecting neurons while HVC neurons projecting to Area X would be stable. It is also likely that HVC interneurons are not replaced in adulthood^13,23,34,59^ (but see^54^ for slightly divergent views relative to developing juvenile zebra finches, *Taeniopygia* *guttata*; reviewed in^11^). It is thus likely that changes in song structure following irradiation reflect a decrease in the pool of HVC neurons projecting to RA. This idea could be studied by combining the present methods with retrograde tracing. If this conclusion was confirmed, it would then be important to analyze how, from a mechanistic point of view, a decreased HVC to RA projection can affect song structure. The irradiation also decreased the number of non-neuronal, likely glial, cells in HVC, thus the possible contribution of this cellular change to the observed behavioral effects should also be considered.

It cannot be excluded at this stage that significant effects detected among a substantial number of unchanged aspects of song only represent a type I statistical error linked to uncontrolled fluctuations in the samples. This appears unlikely based on the fact that the observed difference in entropy and bandwidth only developed progressively after the irradiation and were not present at the beginning of the experiment when the absence of new neurons was presumably minimal. In a previous experiment, the specific depletion of HVC neurons projecting to RA similarly produced limited effects on song structure and this was observed in only a fraction of the birds^23^. It is thus not completely unexpected that behavioral effects observed here were not global but focused. Together, the data suggest that the depletion of HVC neurogenesis induced here was sufficient to affect in a reliable manner specific aspects of song production. This irradiation procedure thus appears to be promising and should be implemented again in future experiments.

### Changes in the activation of auditory areas

HVC is clearly controlling song production^60,61^, but there is evidence that it also plays a role in song auditory discrimination. Song perception in the secondary auditory areas of the telencephalon, NCM and CMM, is associated with an activation of gene transcription as evidenced by an increased expression of the immediate early gene ZENK^47,62,63^ and activity in these areas is modulated by interactions with HVC. Tract-tracing studies have indeed identified indirect connections between the secondary auditory areas and HVC^64–66^.

In addition to its role in the control of female song that is more broadly distributed than initially thought^67,68^, HVC is thus implicated in song perception. For example, females with HVC lesions display copulation solicitation displays (CSD) in response to heterospecific as well as conspecific songs contrary to control females who only respond to conspecific song^18,69^. Accordingly ibotenic acid lesions of HVC drastically decrease ZENK induction in response to male songs in NCM and CMM^70^.

Here, inhibition of neurogenesis in HVC significantly depressed ZENK induction in NCM and CMM in response to male songs playback. Future research based on behavioral techniques should determine whether this differential ZENK activation really reflects a change in auditory perception.

The mechanism that mediated this decrease in ZENK expression remains partly unidentified. The irradiation could somehow have touched also the auditory areas and inhibited the neurogenesis that is known to take place in NCM^71,72^. This interpretation is however not supported by the finding that no decrease of DCX^+^ cells was detected in the three auditory areas considered. In addition, global counts of cells in Nissl-stained sections did not identify a treatment-induced modification. It is thus unlikely that the irradiation that was directed to the dorsal surface of the brain directly affected these auditory areas. The dorsal part of CMM could potentially have been affected but this is impossible for ventral aspects of NCM where a decrease in ZENK induction was also observed.

The decreased ZENK induction must therefore result from a modification of the functional properties of an unchanged number of auditory neurons indirectly induced by the irradiation of HVC. The key question is then whether this effect, as well as the changes in song structure, were directly caused by the decreased neurogenesis or by some other less specific negative impact of the irradiation. A general lesion of HVC might indeed have similar effects (see^70^) but several facts argue against this interpretation. It is useful to recall that the X-rays decreased the number of cells incorporating BrdU as well as the young fusiform DCX^+^ neurons, but not the older multipolar DCX neurons. There was also no change in the volume of HVC. This suggests that effects of irradiation were limited to the progenitor cells and did not affect more mature neurons. We also established that at the individual level, the impaired neurogenesis was correlated with the physiological effects. These data thus strongly argue for effects directly caused by the decline in new neurons incorporation providing for the first time a direct indication of the causal role of adult neurogenesis in HVC. Since new neurons added during adulthood in HVC are mostly or exclusively neurons projecting to RA (see previous section), this would argue that this class of neurons also establishes functional, but probably not anatomical, connections with auditory regions and modulate their responses to sounds and songs in particular. Alternative explanations however remain possible and the neuroanatomical and mechanistic bases of this interaction should now be investigated.

In conclusion, the present data indicate that the irradiation procedure implemented in the present experiments appears as a very promising approach to analyze in a causal manner adult neurogenesis in songbirds and its functional consequences.

## Data Availability

All data generated or analyzed during this study are included as individual data points in the figures of this published article. All these data in numerical format are available and will be provided by the corresponding author in response to any reasonable request.

## References

[1] Garcia-Segura LM (2009). Hormones and Brain Plasticity.

[2] Costandi M (2016). Neuroplasticity.

[3] Gage FH, Kemperman G, Song H (2007). Adult Neurogenesis.

[4] Kemperman G (2011). Adult Neurogenesis. Stem Cells and Neuronal Development in the Adult Brain.

[5] Brenowitz EA, Larson TA (2015). Neurogenesis in the adult avian song-control system. Cold Spring Harbor Perspect. Biol..

[6] Kirn JR, Nottebohm F (1993). Direct evidence for loss and replacement of projection neurons in adult canary brain. J. Neurosci..

[7] Kirn J, O'Loughlin B, Kasparian S, Nottebohm F (1994). Cell death and neuronal recruitment in the high vocal center of adult male canaries are temporally related to changes in song. Proc. Natl. Acad. Sci. USA.

[8] Goldman SA (1998). Adult neurogenesis: From canaries to the clinic. J. Neurobiol..

[9] Nottebohm F (1985). Neuronal replacement in adulthood. Ann. N.Y. Acad. Sci..

[10] Nottebohm, F. *Neuroscience**of**Birdsong* (eds. Zeigler, H.P. & Marler, P.). 425–448 (Cambridge University Press, 2008).

[11] Balthazart J, Ball GF (2016). Endocrine and social regulation of adult neurogenesis in songbirds. Front. Neuroendocrinol..

[12] Nottebohm F, Nottebohm ME, Crane LA, Wingfield JC (1987). Seasonal changes in gonadal hormone levels of adult male canaries and their relation to song. Behav. Neural Biol..

[13] Alvarez-Buylla A, Theelen M, Nottebohm F (1988). Birth of projection neurons in the higher vocal center of the canary forebrain before, during, and after song learning. Proc. Natl. Acad. Sci. USA.

[14] Rasika S, Nottebohm F, Alvarez-Buylla A (1994). Testosterone increases the recruitment and/or survival of new high vocal center neurons in adult female canaries. Proc. Natl. Acad. Sci. USA.

[15] Barker JM, Ball GF, Balthazart J (2014). Anatomically discrete sex differences and enhancement by testosterone of cell proliferation in the telencephalic ventricle zone of the adult canary brain. J. Chem. Neuroanat..

[16] Nottebohm F (1981). A brain for all seasons: Cyclical anatomical changes in song-control nuclei of the canary brain. Science.

[17] Bernard DJ, Eens M, Ball GF (1996). Age- and behavior-related variation in volumes of song control nuclei in male European starlings. J. Neurobiol..

[18] Brenowitz E, Lent K, Kroodsma DE (1995). Brain space for learned song in birds develops independently of song learning. J. Neurosci..

[19] Smith GT, Brenowitz EA, Wingfield JC (1997). Roles of photoperiod and testosterone in seasonal plasticity of the avian song control system. J. Neurobiol..

[20] Ball GF, Riters LV, Balthazart J (2002). Neuroendocrinology of song behavior and avian brain plasticity: Multiple sites of action of sex steroid hormones. Front. Neuroendocrinol..

[21] Cohen RE, Macedo-Lima M, Miller KE, Brenowitz EA (2016). Adult neurogenesis leads to the functional reconstruction of a telencephalic neural circuit. J. Neurosci..

[22] Pytte CL (2016). Adult neurogenesis in the songbird: Region-specific contributions of new neurons to behavioral plasticity and stability. Brain Behav. Evol..

[23] Scharff C, Kirn JR, Grossman M, Macklis JD, Nottebohm F (2000). Targeted neuronal death affects neuronal replacement and vocal behavior in adult songbirds. Neuron.

[24] Hall ZJ, Delaney S, Sherry DF (2014). Inhibition of cell proliferation in black-capped chickadees suggests a role for neurogenesis in spatial learning. Dev. Neurobiol..

[25] Chen G, Cheng MF (2007). Inhibition of lesion-induced neurogenesis impaired behavioral recovery in adult ring doves. Behav. Brain Res..

[26] Yang CF (2013). Sexually dimorphic neurons in the ventromedial hypothalamus govern mating in both sexes and aggression in males. Cell.

[27] Hellier V (2018). Female sexual behavior in mice is controlled by kisspeptin neurons. Nat. Commun..

[28] Johnston S (2021). AAV ablates neurogenesis in the adult murine hippocampus. Elife.

[29] Wojtowicz JM (2006). Irradiation as an experimental tool in studies of adult neurogenesis. Hippocampus.

[30] Lazarini F (2009). Cellular and behavioral effects of cranial irradiation of the subventricular zone in adult mice. PLoS ONE.

[31] Manda K, Ueno M, Anzai K (2009). Cranial irradiation-induced inhibition of neurogenesis in hippocampal dentate gyrus of adult mice: Attenuation by melatonin pretreatment. J. Pineal Res..

[32] Ford EC (2011). Localized CT-guided irradiation inhibits neurogenesis in specific regions of the adult mouse brain. Radiat. Res..

[33] Achanta P (2012). Subventricular zone localized irradiation affects the generation of proliferating neural precursor cells and the migration of neuroblasts. Stem Cells.

[34] Kirn JR, Alvarez-Buylla A, Nottebohm F (1991). Production and survival of projection neurons in a forebrain vocal center of adult male canaries. J. Neurosci..

[35] Kirn JR, Fishman Y, Sasportas K, Alvarez-Buylla A, Nottebohm F (1999). Fate of new neurons in adult canary high vocal center during the first 30 days after their formation. J. Comp. Neurol..

[36] Nottebohm F (1980). Testosterone triggers growth of brain vocal control nuclei in adult female canaries. Brain Res..

[37] Cornez G (2020). Testosterone stimulates perineuronal nets development around parvalbumin cells in the adult canary brain in parallel with song crystallization. Hormones Behav..

[38] Madison FN, Rouse ML, Balthazart J, Ball GF (2015). Reversing song behavior phenotype: Testosterone driven induction of singing and measures of song quality in adult male and female canaries (*Serinus*
*canaria*). Gen. Comp. Endocrinol..

[39] Shevchouk OT, Ghorbanpoor S, Ball GF, Cornil CA, Balthazart J (2017). Testosterone-induced neuroendocrine changes in the medial preoptic area precede song activation and plasticity in song control nuclei of female canaries. Eur. J. Neurosci..

[40] Dos Santos EB, Ball GF, Cornil CA, Balthazart J (2022). Treatment with androgens plus estrogens cannot reverse sex differences in song and the song control nuclei in adult canaries. Hormones Behav..

[41] Cornez G (2021). Perineuronal nets in HVC and plasticity in male canary song. PLoS ONE.

[42] Shevchouk OT (2018). Behavioral evidence for sex steroids hypersensitivity in castrated male canaries. Horm. Behav..

[43] Tchernichovski O, Nottebohm F, Ho CE, Pesaran B, Mitra PP (2000). A procedure for an automated measurement of song similarity. Anim. Behav..

[44] Tchernichovski O, Eisenberg-Edidin S, Jarvis ED (2021). Balanced imitation sustains song culture in zebra finches. Nat. Commun..

[45] Balthazart J, Boseret G, Konkle AT, Hurley LL, Ball GF (2008). Doublecortin as a marker of adult neuroplasticity in the canary song control nucleus HVC. Eur. J. Neurosci..

[46] Boseret G, Ball GF, Balthazart J (2007). The microtubule-associated protein doublecortin is broadly expressed in the telencephalon of adult canaries. J. Chem. Neuroanat..

[47] Mello CV, Vicario DS, Clayton DF (1992). Song presentation induces gene expression in the songbird forebrain. Proc. Natl. Acad. Sci. USA.

[48] Ball GF, Tlemcani O, Balthazart J (1997). Induction of the Zenk protein after sexual interactions in male Japanese quail. NeuroReport.

[49] Mello CV, Ribeiro S (1998). ZENK protein regulation by song in the brain of songbirds. J. Comp. Neurol..

[50] Maney DL, Pinaud R (2011). Estradiol-dependent modulation of auditory processing and selectivity in songbirds. Front. Neuroendocrinol..

[51] Balthazart J, Ball GF (2014). Endogenous versus exogenous markers of adult neurogenesis in canaries and other birds: Advantages and disadvantages. J. Comp. Neurol..

[52] Barker JM, Charlier TD, Ball GF, Balthazart J (2013). A new method for in vitro detection of bromodeoxyuridine in serum: A proof of concept in a songbird species, the canary. PLoS ONE.

[53] Haakenson CM, Madison FN, Ball GF (2019). Effects of song experience and song quality on immediate early gene expression in female canaries (*Serinus*
*canaria*). Dev. Neurobiol..

[54] Scott BB, Lois C (2007). Developmental origin and identity of song system neurons born during vocal learning in songbirds. J. Comp. Neurol..

[55] Vellema M, van der Linden A, Gahr M (2010). Area-specific migration and recruitment of new neurons in the adult songbird brain. J. Comp. Neurol..

[56] Alvarez-Buylla A, Theelen M, Nottebohm F (1990). Proliferation, "hot spots" in adult avian ventricular zone reveal radial cell division. Neuron.

[57] Cornez G (2020). Development of perineuronal nets during ontogeny correlates with sensorimotor vocal learning in canaries. eNeuro..

[58] Alward BA, Balthazart J, Ball GF (2017). Dissociable effects on birdsong of androgen signaling in cortex-like brain regions of canaries. J. Neurosci..

[59] Scotto-Lomassese S, Rochefort C, Nshdejan A, Scharff C (2007). HVC interneurons are not renewed in adult male zebra finches. Eur. J. Neurosci..

[60] Nottebohm F, Stokes TM, Leonard CM (1976). Central control of song in the canary, *Serinus*
*canarius*. J. Comp. Neurol..

[61] Margoliash D (1997). Functional organization of forebrain pathways for song production and perception. J. Neurobiol..

[62] Clayton DF (1997). Role of gene regulation in song circuit development and song learning. J. Neurobiol..

[63] Ribeiro S, Cecchi GA, Magnasco MO, Mello CV (1998). Toward a song code: Evidence for a syllabic representation in the canary brain. Neuron.

[64] Bauer EE (2008). A synaptic basis for auditory-vocal integration in the songbird. J. Neurosci..

[65] Akutagawa E, Konishi M (2010). New brain pathways found in the vocal control system of a songbird. J. Comp. Neurol..

[66] Theunissen, F. E. *et**al.**Neuroscience**of**Birdsong* (eds. Ziegler, P. H. & Marler, P.). 157–173 (Cambridge Univerity Press, 2008).

[67] MacDougall-Shackleton SA, Ball GF (1999). Comparative studies of sex differences in the song-control system of songbirds. Trends Neurosci..

[68] Odom KJ, Hall ML, Riebel K, Omland KE, Langmore NE (2014). Female song is widespread and ancestral in songbirds. Nat. Commun..

[69] Del Negro C, Gahr M, Leboucher G, Kreutzer M (1998). The selectivity of sexual responses to song displays: effects of partial chemical lesion of the HVC in female canaries. Behav. Brain Res..

[70] Lynch KS, Kleitz-Nelson HK, Ball GF (2013). HVC lesions modify immediate early gene expression in auditory forebrain regions of female songbirds. Dev. Neurobiol..

[71] Tsoi SC (2014). Hemispheric asymmetry in new neurons in adulthood is associated with vocal learning and auditory memory. PLoS ONE.

[72] Aronowitz JV (2021). Unilateral vocal nerve resection alters neurogenesis in the avian song system in a region-specific manner. PLoS ONE.

